# Investigating the Neural Basis of Theta Burst Stimulation to Premotor Cortex on Emotional Vocalization Perception: A Combined TMS-fMRI Study

**DOI:** 10.3389/fnhum.2018.00150

**Published:** 2018-05-15

**Authors:** Zarinah K. Agnew, Michael J. Banissy, Carolyn McGettigan, Vincent Walsh, Sophie K. Scott

**Affiliations:** ^1^Institute of Cognitive Neuroscience, University College London, London, United Kingdom; ^2^Otolaryngology-Head & Neck Surgery Clinic, University of California, San Francisco, San Francisco, CA, United States; ^3^Department of Psychology, Goldsmiths, University of London, London, United Kingdom; ^4^Royal Holloway, University of London, Egham, United Kingdom

**Keywords:** cTBS, transcranial magnetic stimulation, emotional vocalization, emotions, premotor cortex, functional magnetic resonance imaging, fMRI

## Abstract

Previous studies have established a role for premotor cortex in the processing of auditory emotional vocalizations. Inhibitory continuous theta burst transcranial magnetic stimulation (cTBS) applied to right premotor cortex selectively increases the reaction time to a same-different task, implying a causal role for right ventral premotor cortex (PMv) in the processing of emotional sounds. However, little is known about the functional networks to which PMv contribute across the cortical hemispheres. In light of these data, the present study aimed to investigate how and where in the brain cTBS affects activity during the processing of auditory emotional vocalizations. Using functional neuroimaging, we report that inhibitory cTBS applied to the right premotor cortex (compared to vertex control site) results in three distinct response profiles: following stimulation of PMv, widespread frontoparietal cortices, including a site close to the target site, and parahippocampal gyrus displayed an increase in activity, whereas the reverse response profile was apparent in a set of midline structures and right IFG. A third response profile was seen in left supramarginal gyrus in which activity was greater post-stimulation at both stimulation sites. Finally, whilst previous studies have shown a condition specific behavioral effect following cTBS to premotor cortex, we did not find a condition specific neural change in BOLD response. These data demonstrate a complex relationship between cTBS and activity in widespread neural networks and are discussed in relation to both emotional processing and the neural basis of cTBS.

## Highlights

• Premotor cortex plays a causal role in auditory emotional vocalization processing.
• We use fMRI to investigate neural activity before and after cTBS to premotor cortex.
• cTBS elicits changes in BOLD activity in widespread regions including the target site.
• Inhibitory cTBS results in both increases and decreases in BOLD responses.
• No condition specific effect of cTBS on neural activity, despite a specific behavioral effect.

## Introduction

The last two decades have provided increasing evidence that the adult brain is highly susceptible to short-term changes, both in structure and function. Widespread changes in brain organization can occur as a result of both damage or learning and development, over a range of timescales. Studies in humans and animals have demonstrated that neuroplasticity is not restricted to any given developmental phase but remains an ongoing process in the form of learning. In humans, the advent of transcranial magnetic stimulation (TMS) for non-invasive brain stimulation had rendered the human brain accessible for *in vivo* investigation of the neural changes associated with behavioral changes. Here we assess the effect of theta burst TMS on neural activity in a previously established emotional vocalization perception paradigm.

Understanding the emotional states of others is a pivotal aspect of our social interactions. Vocal cues are one of several sources of information that contribute to this ability, with changes in a variety of acoustic cues (e.g., pitch, intensity) providing a rich source of information about the emotional states of others (Sauter, 2010; Sauter et al., 2010). In recent years, a number of brain imaging studies have begun to delineate neural regions that contribute to the perception and appraisal of affective vocalizations, implicating a widespread brain network in this process including the posterior superior temporal gyrus (pSTG), posterior middle temporal gyrus (pMTG), the inferior frontal gyrus (IFG), ventral premotor cortex (PMv), and amygdala (Wildgruber et al., 2005; Warren et al., 2006; Meyer et al., 2007; Paulmann et al., 2008; Wiethoff et al., 2009; Leitman et al., 2010; Ethofer et al., 2012). In addition, lesion and non-invasive brain stimulation studies have demonstrated that disrupting neural activity in specific aspects of this network results in impairments in the appraisal of vocal emotions (Hornak et al., 2003; Rymarczyk and Grabowska, 2007; Hoekert et al., 2008; Banissy et al., 2010). Studies have further shown that whilst TMS can elicit plasticity, there are also a range of factors that contribute to high variability in the neural and behavioral responses to stimulation (Ridding and Ziemann, 2010; Hamada et al., 2013). Thus understanding the neural basis of stimulation induced behavioral changes is of great relevance.

We recently showed that continuous theta burst stimulation (cTBS) targeted at right PMv (rPMv) disrupts the ability to discriminate between emotional vocalizations, but not speaker identity (Banissy et al., 2010). Continuous TBS is an offline form of transcranial magnetic stimulation, in which short bursts of low intensity, high frequency magnetic pulses leads to suppression of cortical activity for up to 1 h (Di Lazzaro et al., 2005; Huang et al., 2005). The method has been used in several domains to demonstrate the involvement of specific cortical regions in cognitive and perceptual tasks (Vallesi et al., 2007; Kalla et al., 2009; Rounis et al., 2010; Verbruggen et al., 2010) and is becoming a prominent tool in non-invasive brain stimulation studies of cognition. While of clear utility in demonstrating the degree of involvement of a brain region in a given task, the global effects that result from cTBS are unclear, and little is known about how stimulation to a region may influence processing across a functional network (Walsh and Pascual-Leone, 2003).

### Effects of rTMS

Recent work has shown that repetitive TMS (rTMS) can elicit changes in a number of different ways: Zanto et al. (2013) report that rTMS elicits changes in task-related brain regions without affecting behavior, on the other hand, studies have also showed that stimulation of intraparietal cortex modulates activity in visual cortex in a context specific manner (Ruff et al., 2008). In addition to this, others have reported changes to activity in the contralateral homolog of stimulation site, with interhemispheric connectivity being correlated with increased performance (Andoh and Zatorre, 2012). Ward et al. (2010) showed that rTMS to dorsal premotor cortex (PMd) resulted in reduced activity at the target site, but increased task related activity coupling with other regions. Other studies have confirmed that repetitive TMS results in local and distributed changes to neural processing (Binney and Ralph, 2015; Hallam et al., 2016).

### Effects of cTBS

Continuous TBS has also been shown to alter activity in functionally connected but distinct sites from the site of stimulation (Ott et al., 2011; Jung and Lambon Ralph, 2016). For example, in relation to speech, cTBS has been used to suppress activity in a focal region, which resulted in increased activity in the right homolog region (Hartwigsen et al., 2013). These studies have largely provided evidence that cTBS results in reduction of activity at the site of stimulation, and an upregulation of activity in other distinct regions, comprising homologous sites and neighboring regions. These data have been interpreted as a reflection of compensatory effects of a disrupted neural network in order to attempt to maintain function.

Here, we sought to utilize and assess the network effects of cTBS based on these previous findings, by combining it with fMRI in order to investigate how the application of cTBS to rPMv influences BOLD responses to emotional sounds (i) across the whole brain, (ii) in regions activated by emotional vocalization perception, (iii) at the target site and finally (iv) at the left hemisphere homolog of the target site. Specifically, we compared BOLD responses during passive listening of different emotional vocalizations (as per Warren et al., 2006) before and after cTBS targeted at the rPMv or the vertex (active control stimulation site). Given our previous findings highlighting the role of rPMv as part of a network of regions involved in the passive perception of emotional vocalizations (Warren et al., 2006) and the cTBS findings of us and others (Banissy et al., 2010) (see above), we predicted that we would see changes to activity within the emotional sound processing network. Based on previous findings (Jung and Lambon Ralph, 2016) and others, we expected to see a reduction in activity at the site of stimulation and sought to explore the nature of more disparate neural effects.

## Materials and Methods

The experimental set up required the following conditions: Passive perception of sounds of (1) amusement, (2) triumph, (3) fear, (4) disgust, (5) spectrally rotated versions of a mixture of all four emotional sounds and (6) a silent rest condition (see below for details). Subjects were required to attend two sessions on different days. On each visit subjects underwent two 20-min fMRI experiments which involved the six experimental conditions as listed above. Following the first 20-min fMRI experiment, subjects then underwent cTBS to either the rPMv or to the vertex (the order of sessions were counterbalanced across participants), before going back into the scanner to repeat the fMRI experiment. On the second visit, subjects underwent the same procedure but had cTBS applied to the other stimulation site. At least a week was left between the active and control site stimulation sessions.

### Auditory Stimuli

Four different types of emotional vocalizations were used, two positive (amusement, triumph) and two negatively valenced (fear, disgust) (Ekman, 1992, 2003). The stimuli were developed and employed in a number of previous studies (Warren et al., 2006; Banissy et al., 2010; Sauter, 2010). The baseline stimuli were spectrally rotated versions (Blesser, 1972; Scott et al., 2000) of the experimental stimuli. This manipulation involves equalizing each of the stimuli with a high pass filter. This manipulation affords the rotated signal approximately the same long-term spectrum as the original. The equalized signal is then amplitude-modulated by sinu-manipulation to produce unintelligible sounds that lack the human vocal quality of the original stimuli but maintain a comparable level of acoustic complexity (Blesser, 1972). All auditory stimuli were scaled to the same peak amplitude. Each rotated trial was composed of four sounds played successively.

### Subjects

Sixteen healthy right-handed subjects (range 23–49 years) participated in the study. All gave informed consent according to the guidelines approved by UCL Ethics Committee, who provided local ethics approval for this study.

### Functional MRI

Functional imaging data were acquired on a 1.5 Tesla Siemens Avanto system (Siemens AG, Erlangen, Germany) using a 32-channel birdcage head coil. This type of head coil has been shown to increase signal-to-noise for medical images acquired in the 1.5 Tesla field without increasing the signal drop out associated with higher field strengths (Fellner et al., 2009; Parikh et al., 2011). T_2_^∗^-weighted echo-planer images (EPI) were acquired (3 mm × 3 mm × 3 mm, TR/TA/TE/flip 10,000 ms/3 s/50 ms/90°) using BOLD contrast. On each day, subjects were scanned twice, pre- and post- stimulation. On each day of scanning, 250 volumes were acquired in two runs – 125 volumes in the pre-stimulation run and 125 in the post-stimulation run. Subjects were required to come in on two separate days for two separate scanning sessions, during which time they received stimulation to either the vertex or right PMv. In all cases, the first two functional volumes were discarded in order to remove the effect of T_1_ equilibration. High-resolution T_1_ anatomical volume images (160 sagittal slices, voxel size 1 mm^3^) were also acquired for each subject.

During the main experimental run, subjects lay supine in the scanner in the dark and were asked to close their eyes and listen to the sounds played to them. There was no task involved so as to avoid any form of motor priming that a response, such as a button press, or a cognitive task might entail. Functional data were acquired using a sparse sampling protocol in which four stimuli were presented during the quiet 7 s intervals (±500 ms jitter) between image acquisitions. This approach allowed the presentation of auditory stimuli in the absence of interference from scanner noise (Hall et al., 1999). Stimuli were presented using matlab (Mathworks, version 7.10) using the Psychophysics Toolbox extension (Brainard, 1997).

Sounds were presented to the subjects in the scanner via a Denon amplifier (Denon UK, Belfast, United Kingdom) and air conduction headphones (Etymotic Inc., Elk Grove Village, IL, United States) worn by the subjects. During each run, subjects heard stimuli from one of each of the five experimental conditions (four per trial). There were 20 examples of each condition within each session (pre- and post-TBS) played in a randomized order with ±500 ms onset jitter. The same items were not heard more than once in each run. For the high-level baseline condition, four spectrally rotated versions were played. These were a combination of spectrally rotated versions of all four classes of emotional vocalization. For the silent rest condition subjects heard nothing during the silent period between image acquisitions. The presentation of stimuli was pseudo-randomized to allow a relatively even distribution of the six conditions in the absence of order effects.

Between the two 20 min fMRI sessions, subjects were brought out of the scanner in order to administer cTBS. Following application of cTBS subjects were instructed not to speak unless they had to, and were each put back into the scanner immediately. The beginning of the second scanning session was timed such that the functional data acquisition began 5 min after the end of the cTBS.

### TMS Parameters and Co-registration

Transcranial magnetic stimulation was delivered via a figure of eight coil with a 70 mm diameter using a Magstim Super Rapid Stimulator (Magstim, United Kingdom) at 40% machine output. This intensity is in accordance with other cTBS studies (Mochizuki et al., 2005; Koch et al., 2008; Nyffeler et al., 2008, 2009; Mensen et al., 2014) and studies that have employed fixed intensities above 80% motor threshold when combining cTBS with fMRI (Pitcher et al., 2014, 2017).

An offline cTBS paradigm was used, which consisted of a burst of 3 pulses at 50 Hz repeated at intervals of 200 ms for 20 s, resulting in a total of 300 pulses. Based upon previous findings (Di Lazzaro et al., 2005; Huang et al., 2005) the time window of reduced excitability following theta burst stimulation was expected to last between 20–30 min and a 5 min rest period after stimulation offset was implemented for each site stimulated.

Locations for cTBS were identified using Brainsight TMS-magnetic resonance co-registration system (Rogue Research, Montreal, QC, Canada). FSL software (FMRIB, Oxford) was used to transform coordinates for each site to each subject’s individual MRI scan. The coordinates for rPMv [54, -2, 44] were the averages of neurologically normal participants in an fMRI study of non-verbal auditory emotion processing (Warren et al., 2006) found to be modulated by emotional category and also active during a motor localiser. These same coordinates were used in our prior cTBS study of emotion vocalization discrimination (Banissy et al., 2010). The control site used was vertex, identified as the point midway between the inion and the nasion, equidistant from the left and right intertragal notches.

### Pre-processing and Analyses

Functional data were analyzed using SPM8 (Wellcome Department of Imaging Neuroscience, London, United Kingdom) running on Matlab 7.4 (Mathworks Inc., Sherborn, MA, United States). All functional images were realigned to the first volume by six-parameter rigid body spatial transformation. Functional images were normalized into standard space using the Montreal Neurological Institute (MNI) template using parameters elicited from a unified segmentation of the T1 anatomical image. Functional images were then smoothed using a Gaussian kernel of full width half medium (FWHM) 8 mm.

**FIGURE 1:**
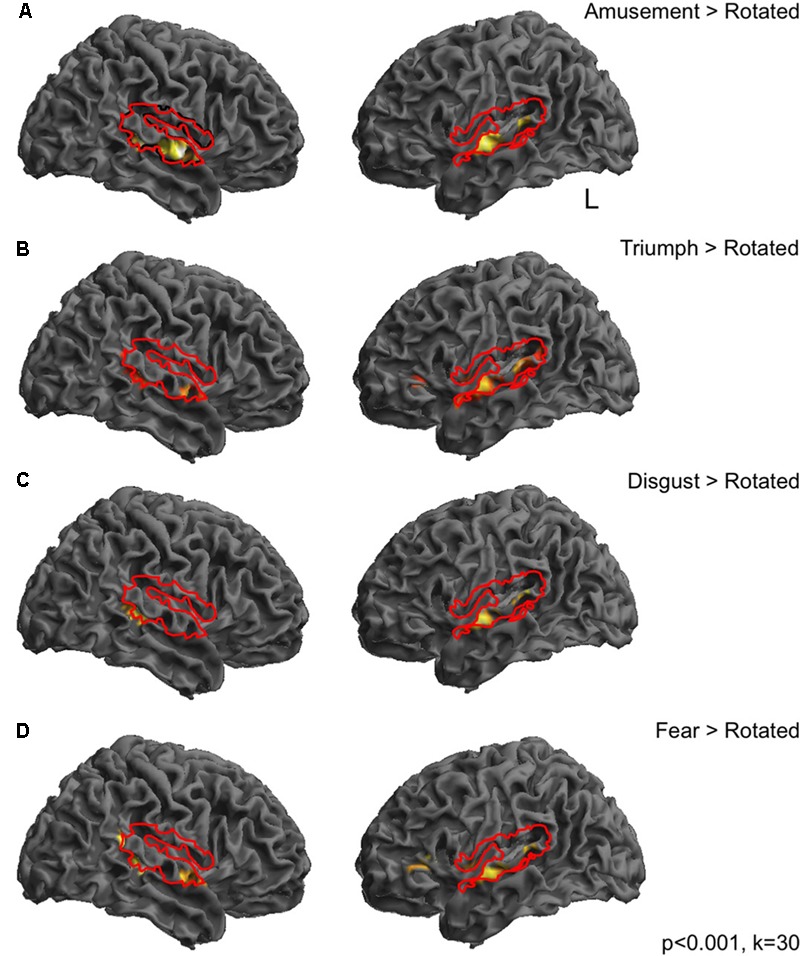
Responses during passive perception of emotional vocalizations in dorsolateral temporal cortices. Bold responses to emotional sounds compared to spectrally rotated versions were seen in middle and posterior superior temporal gyri in both hemispheres for all categories of emotional sound, largely lying within cortical regions revealed by the comparison of all sounds compared to rest (depicted by solid red lines). In most cases responses were more distributed in the left hemisphere **(A–D)**. Perception of triumph and fear sounds was associated with an additional peak in the left inferior frontal gyrus **(B,D)**. Thresholded at *p* < 0.001 uncorrected with a cluster extent threshold of 30 voxels.

**FIGURE 2:**
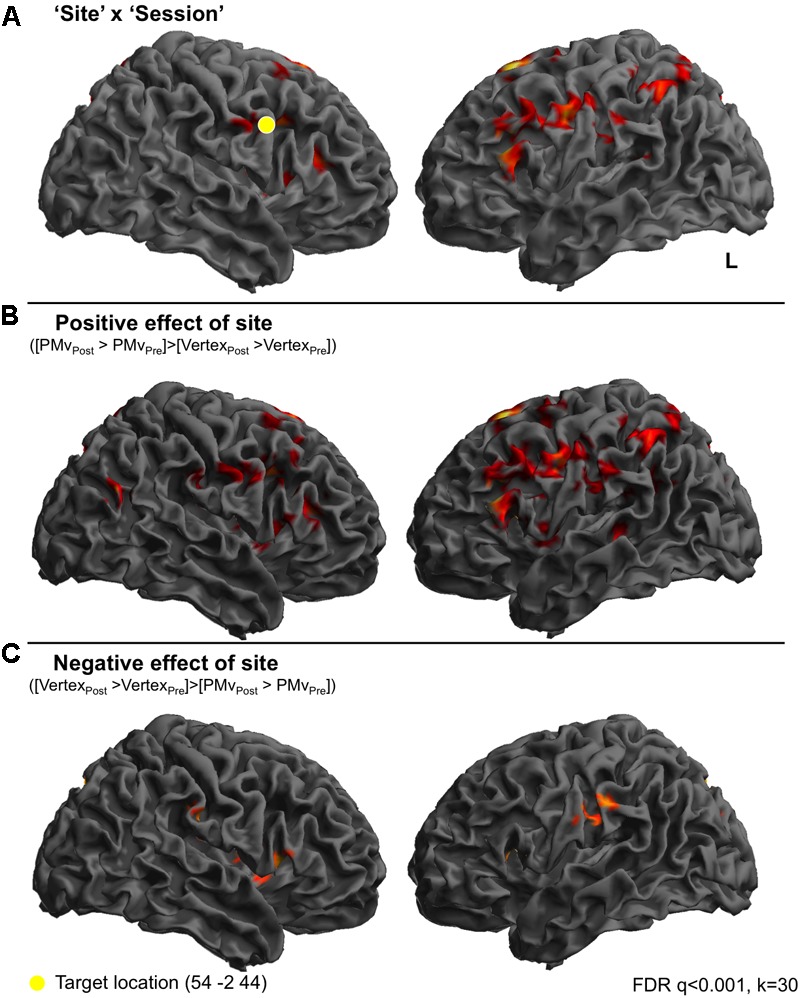
Continuous TBS to premotor cortex increases BOLD responses in frontoparietal cortices compared to vertex stimulation. In order to look at the effect of cTBS to right premotor cortex on perception of sounds, compared to stimulation at the vertex, individual *t*-tests for the comparison of each condition post-stimulation > pre-stimulation were calculated. These *t*-tests, which reflect the difference between pre- and post-stimulation were then entered into a 2 × 6 ANOVA, whereby the two factors were ‘Site’ (premotor and vertex) and ‘Condition’ (Amusement, Disgust, Fear Triumph, Rotated, Rest). These *t* contrasts reflect the difference between pre- and post-stimulation, and such that effects of the Site and Condition factors in fact reflect the interaction of these factors with the effect of Session (pre- vs. post-stimulation). A ‘Site’ × ‘Session’ interaction was observed in inferior frontal gyri, left middle frontal gyrus and right insula cortex and finally in right precentral gyrus. A second set of clusters lay in bilateral inferior parietal cortex and postcentral gyrus on the right, left supplementary motor area extending into superior frontal gyrus. Finally a set of midline clusters were seen in right middle cingulate cortex, precuneus and parahippocampal gyrus. **(A)** A *t*-test revealed greater activity for the comparison of PMv post-stimulation compared to PMv pre-stimulation, than vertex post-stimulation compared to vertex pre-stimulation, in left inferior frontal gyrus, left supplementary motor area, cerebellar vermis, right parahippocampal gyrus, bilateral inferior parietal lobe, bilateral superior and right middle frontal gyri and right postcentral gyrus **(B)**. The opposite comparison was associated with activity in left hippocampus, right middle cingulate cortex, right precuneus, left supramarginal gyrus, right supplementary motor area, right inferior frontal gyrus and right rolandic operculum **(C)**.

Event-related responses for each condition were modeled as a canonical hemodynamic response function. Event onsets were modeled from the onset of auditory recording with a 4 s duration. For each run (pre- and post- stimulation) each condition was modeled as a separate regressor in a general linear model. Motion parameters (three translations and three rotations) were modeled as six regressors of no interest at the single-subject level. For each subject (first level), contrast images were created to describe the comparisons between each of the experimental conditions compared to silent rest and to each other. *T*-tests were also generated comparing individual conditions pre- and post-stimulation for each site of stimulation. The contrast images generated from these *t*-tests were entered into group analyses at the second level. Peak activations were localized using the anatomy toolbox available within SPM8 (Eickhoff et al., 2005). In the case of basic contrasts (**Figure 1**) with clear anatomical hypotheses that activity would be observed in superior dorsolateral temporal cortices, statistical maps were thresholded at *p* < 0.001 with a cluster extent of 30 voxels. In the case of more exploratory comparisons (**Figure 2**, onward), FDR correction for multiple comparisons was applied (*q* < 0.001, *k* = 30).

Data were analyzed in a number of different ways:

(1) In order to investigate BOLD responses to perception of emotional sounds, the two pre-stimulation sessions were combined into one model, with the 2 days being modeled as separate sessions within one design matrix. Contrasts were then created at the first level of each of the sound categories compared to silent rest and compared to the high-level baseline of rotated sounds. Resulting first level contrasts were entered into *t*-tests at the second level.
(2) In order to investigate the effect of stimulation to either premotor and vertex sites on the neural activity during perception of emotional sounds, contrasts were generated at the first level, comparing perception of each emotional vocalization category post-stimulation with pre-stimulation (e.g., Amusement_[post-stim]_ > Amusement_[pre-stim]_). This was performed separately for premotor stimulation and vertex stimulation data. In order to account for within subject variation, these contrasts were then taken up to a second level 2 × 6 fully flexible ANOVA where the two factors were Site (Premotor and Vertex) and Condition (Amusement_[post>pre]_, Triumph_[post__>__pre]_, Disgust_[post>pre]_, Fear_[post>pre]_, Rotated_[post>pre]_, and Rest_[post>pre]_). In this way, the contrasts entered into the ANOVA at the second level reflect the difference between pre- and post-stimulation, and so any observed effects of the Site and Condition factors within this model in fact reflect the interaction of these factors with the effect of Session (post- vs. pre-stimulation).
(3) Region of interest analyses were carried out to investigate mean effect sizes in specific regions across all experimental conditions post-stimulation compared to pre-stimulation using the MarsBar toolbox that is available for use within SPM8 (Brett et al., 2002).

### First Level Contrasts of Interest

Amusement > Silent Rest/Amusement > Spectrally rotated/Triumph > Silent Rest/Triumph > Spectrally rotated/Fear > Silent Rest/Fear > Spectrally rotated/Disgust > Silent Rest/Disgust > Spectrally rotated/Amusement_[post-stim]_ > Amusement_[pre-stim]_) [PMv site]/Triumph_[post-stim]_ > Triumph_[pre-stim]_) [PMv site]/Fear_[post-stim]_ > Fear_[pre-stim]_) [PMv site]/ Disgust_[post-stim]_ > Disgust_[pre-stim]_) [PMv site]/Amusement_[post-stim]_ > Amusement_[pre-stim]_) [Vertex site]/Triumph_[post-stim]_ > Triumph_[pre-stim]_) [Vertex site]/Fear _[post-stim]_ > Fear_[pre-stim]_) [Vertex site]/Disgust_[post-stim]_ > Disgust_[pre-stim]_) [Vertex site] (see **Table 1** for anova set up).

**Table 1:** 2 × 6 way ANOVA.

Factor 1. Condition	Factor 2. Stimulation Site	
	Pre motor cortex (PMv)	Vertex
	Triumph_[post-stim]_ > Triumph_[pre-stim]_)	Triumph_[post-stim]_ > Triumph_[pre-stim]_)
	Fear_[post-stim]_ > Fear_[pre-stim]_)	Fear_[post-stim]_ > Fear_[pre-stim]_)
	Disgust_[post-stim]_ > Disgust_[pre-stim]_)	Disgust_[post-stim]_ > Disgust_[pre-stim]_)
	Amusement_[post-stim]_ > Amusement_[pre-stim]_)	Amusement_[post-stim]_ > Amusement_[pre-stim]_)

## Results

### Basic Contrasts (*p* < 0.001, *k* = 30)

In order to investigate BOLD responses to perception of emotional sounds, the two pre-stimulation sessions were combined into one model. Contrasts were then created at the first level of each of the sound categories compared to silent rest and compared to the high-level baseline of rotated sounds. Perception of emotional sounds pre-stimulation, compared to a silent rest condition was associated with widespread significant activity in the dorsolateral temporal cortices in both hemispheres (**Figure 1**, red lines). Perception of each emotional vocalization compared to the spectrally rotated sounds (i.e., controlling for temporal and spectral complexity) was associated with significant BOLD responses in a more restricted pattern comprising peaks in middle and posterior superior temporal gyri in both hemispheres (**Figures 1A–D**). In the cases of Fear and Triumph, peaks were also seen in left inferior frontal gyrus (**Figures 1B,D**). These initial contrasts were thresholded at *p* < 0.001 with a cluster threshold of 30 voxels.

### Comparisons Post- and Pre-stimulation (FDR *q* < 0.001, *k* = 30)

We next addressed whether post- vs. pre-stimulation differences were significantly different between the target sites. In order to look at the different post-stimulation compared to pre-stimulation in the premotor site compared to the vertex site, contrasts such as [Amusement_[post-stim]_ > Amusement_[pre-stim]_] reflecting the difference between pre- and post-stimulation, were entered into an ANOVA at the second level. Observed effects of the Site and Condition factors within this model in fact reflect the interaction of these factors with the effect of Session (pre- vs. post-stimulation). We aimed to look at how cTBS modulated BOLD responses (i) across the whole brain, (ii) in regions activated by emotional vocalization perception, (iii) at the target site and finally (iv) at the left hemisphere homolog of the target site (see **Table 2** for a list of coordinates).

**Table 2:** Significant peaks of BOLD activity in contrasts of interest.

*p*	*z* score	*k*	*x*	*y*	*z*
**Main effects condition**					
**Amusement > Rotated (*p* > 0.001, *k* = 30)**					
0.009	4.45	122	63	-4	-8
	4.03		57	-16	1
	3.88		66	-13	-2
	4.34	96	-60	-13	-2
	3.58		-57	-1	-5
	4.14	33	45	-28	4
**Disgust > Rotated (*p* > 0.001, *k* = 30)**					
0.004	4.29	60	48	-34	4
	3.34		57	-22	-2
	3.34		63	-16	-2
	4.26	32	-63	-37	10
	4.09	58	-60	-13	-5
	3.42		-54	5	-8
**Fear > Rotated (*p* > 0.001, *k* = 30)**					
0	4.49	99	45	-31	1
	3.91		57	-40	16
	3.49		45	-43	19
	4.06	76	-57	-4	-2
	3.94		-54	-22	-2
	3.99	48	-63	-37	10
	3.93	38	-48	20	4
	3.49		-48	35	1
	3.49	24	57	-7	-5
	3.38		57	2	-8
**Triumph > Rotated (*p* > 0.001, *k* = 30)**					
0	5.22	139	-54	-10	-2
	3.93		-54	5	-8
	5.05	104	-60	-34	10
	4.14		-51	-49	19
	4.78	70	-42	26	1
	4.43	31	60	-4	-8
	4.14	111	48	-31	4
	3.83		57	-40	16
	3.49		60	-22	-2
**ANOVA**					
**Site × Session (FDR corrected, *q* > 0.001, *k* = 30)**					
0	Inf	70	-30	-40	10
	5.35		-24	-52	19
	4.91		-27	-61	10
	6.76	33	-21	-25	22
	5.57		-18	-40	25
	6.36	45	9	11	34
	4.67		15	17	31
	6.19	51	9	-46	19
	4.58		15	-52	13
	5.58	31	9	-16	28
	3.7		-3	-19	28
	5.37	69	-51	-31	31
	4.42		-57	-19	31
	3.49		-48	-25	25
	5.08	33	15	-28	49
	4.47		15	-46	40
	4.72	101	48	20	4
	4.71		42	14	7
	4.63		39	5	10
	4.56	176	6	-76	46
	4.48		-6	-73	43
	4.23		-18	-55	25
	4.34	35	45	-19	19
	4.3		57	-25	28
**Positive effect of site (FDR corrected, *q* > 0.001, *k* = 30)**					
0	Inf	497	-45	26	22
	6.33		-51	26	13
	6.09		-48	8	28
	7.19	165	-6	20	58
	4.92		-18	17	49
	4.78		-9	20	43
	6.54	32	3	-46	-5
	5.33		-9	-43	-5
	3.65		-18	-40	-8
	6.02	40	27	-40	-5
	5.91	32	54	-64	25
	5.82	243	-42	-49	46
	5.74		-48	-49	58
	4.76		-42	-61	55
	5.31	45	-24	2	70
	4.74		-18	2	55
	5.29	44	39	32	16
	4.95	31	-3	-88	22
	4.7	55	66	-10	31
	4.69		57	2	34
	4.39	39	27	11	61
**Negative Effect of Site (FDR corrected, *q* > 0.001, *k* = 30)**					
0	Inf	70	-30	-40	10
	5.35		-24	-52	19
	4.91		-27	-61	10
	6.76	33	-21	-25	22
	5.57		-18	-40	25
	6.36	45	9	11	34
	4.67		15	17	31
	6.19	51	9	-46	19
	4.58		15	-52	13
	5.58	31	9	-16	28
	3.7		-3	-19	28
	5.37	69	-51	-31	31
	4.42		-57	-19	31
	3.49		-48	-25	25
	5.08	33	15	-28	49
	4.47		15	-46	40
	4.72	101	48	20	4
	4.71		42	14	7
	4.63		39	5	10
	4.56	176	6	-76	46
	4.48		-6	-73	43
	4.23		-18	-55	25
	4.34	35	45	-19	19
	4.3		57	-25	28

*Peak coordinates of significant clusters are reported in **Table 1**, with corresponding z scores, cluster size and mni coordinates. Main comparisons for pre-stimulation emotional vocalizations sessions are compared with spectrally rotated sounds, and interactions between ‘Site’ and ‘Session,’ main positive and negative effects of ‘Site’ are shown.*

At the first level, contrasts were made comparing perception of each emotional vocalization category post-stimulation with pre-stimulation (e.g., Amusement post-stimulation > Amusement pre-stimulation). This was performed separately for premotor and vertex stimulation data sets. These contrasts were then taken up to a second level 2 × 6 fully factorial ANOVA with two factors ‘Site’ (premotor and vertex) and ‘Condition_(post__>__pre)_’ (Amusement_[post>pre]_, Triumph_[post>pre]_, Disgust_[post>pre]_, Fear_[post>pre]_, Rotated_[post>pre]_ and Rest_[post>pre]_). A [Session × Site] interaction was seen in a network of frontal, parietal and midline regions: one bilateral set of clusters lay in inferior frontal gyri with peaks in pars triangularis (BA45) and subpeaks in pars opercularis (BA44), left middle frontal gyrus and right insula cortex and finally in right precentral gyrus (BA6) corresponding to premotor cortex. A second set of clusters lay in bilateral parietal cortex comprising inferior parietal cortex and supramarginal gyrus on the left and postcentral gyrus (BA1) on the right, left supplementary motor area extending into superior frontal gyrus. Finally, a set of midline clusters was seen in right middle cingulate cortex, precuneus and parahippocampal gyrus (**Figure 2A**). **Figure 2B** displays a t-contrast that reflects the positive difference between post- and pre-stimulation sessions for the two sites ([PMv_post_ > PMv_pre_] > [Vertex_post_ > Vertex_pre_]). This comprised left inferior frontal gyrus encompassing both *pars opercularis* and *triangularis*, left supplementary motor area, cerebellar vermis, right parahippocampal gyrus, bilateral inferior parietal lobe encompassing the angular gyrus, bilateral superior and right middle frontal gyri and right postcentral gyrus (**Figure 2B**). The opposite comparison was associated with activity in left hippocampus, right middle cingulate cortex, right precuneus, left supramarginal gyrus, right supplementary motor area, right inferior frontal gyrus (*pars triangularis*) and right rolandic operculum (**Figure 2C**).

**FIGURE 3:**
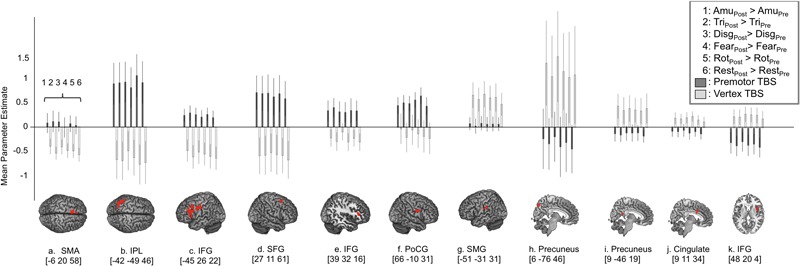
Region of interest analysis reveals three distinct response profiles in response to cTBS to premotor cortex. In order to look at the response profiles of the regions revealed by the ‘Site’ × ‘Session’ interaction, mean parameter estimates were extracted from each cluster and are displayed in **(A–K)** where positive values indicate greater activity post-stimulation compared to pre-stimulation (i.e., increased activity following cTBS) and negative values indicate a reduction in activity following cTBS. This approach revealed one network of regions that show increased levels of activity following stimulation to right PMv including supplementary motor area, inferior parietal cortex, bilateral inferior frontal gyrus, superior frontal gyrus and post central gyrus lying close to the PMv target site (graphs **A–F**). In contrast to this, two distinct regions of the precuneus, cingulate cortex and right IFG displayed a reduction in activity following stimulation to premotor cortex (graphs **H–K**) and less activity following premotor stimulation compared to following vertex stimulation. A third and final response profile was observed in a single cluster in left supramarginal gyrus whereby activity was greater post-stimulation at both sites, compared to pre-stimulation **(G)**.

In order to look at how BOLD responses were modulated by cTBS at specific cortical sites, region of interest analyses were carried out to investigate mean effect sizes in specific regions across all experimental conditions post-stimulation compared to pre-stimulation. In order to look specifically in regions activated by emotional vocalization perception we plotted mean parameter estimates in regions revealed by the interaction [Session × Site], mean parameter estimates were extracted for each of the clusters and are plotted in **Figure 3**. Here, a value of zero would indicate no change in activity levels between pre- and post-stimulation. Positive values on the graph indicate that activity was greater post-stimulation than pre-stimulation and vice versa. Similarly, smaller values for contrasts comparing post- to pre-stimulation following premotor stimulation (gray bars) than following vertex stimulation (white bars) indicates that premotor stimulation resulted in a greater reduction in activity. These plots reveal three important factors: first, the effect of stimulation to vertex was considerable and was variable across regions in terms of direction and extent. In roughly half of the regions, activity was greater post vertex stimulation and in the other half activity was less post vertex stimulation. Given that stimulation at the vertex did alter activity in a number of regions, a more meaningful comparison is between the effect post-PMv stimulation to the effect post-vertex stimulation. The second point that is evident from these plots is the presence of three different patterns of activity within this larger network of clusters. A set of regions comprising bilateral IFG and parietal cortices, left SMA, right superior frontal gyrus and parahippocampal gyrus, displayed an increase in activity post-stimulation to PMv but a decrease in activity post vertex stimulation (**Figures 3A–F**). Secondly, the reverse response profile is apparent in precuneus, middle cingulate cortex and right IFG (**Figures 3H–K**). Finally a single cluster lying over the left supramarginal gyrus displayed a distinct activity profile whereby activity was greater post-stimulation at both sites, compared to pre-stimulation (**Figure 3G**). Lastly, it is evident from these plots that the effect of cTBS to PMv is affecting activity in these regions across all conditions, including during listening to the high level baseline of rotated sounds and during silent rest trials, not just during the perception of emotional vocalizations. Thus the result that we report is not specific to emotional vocalization perception but is a generalized neural effect of cTBS.

**FIGURE 4:**
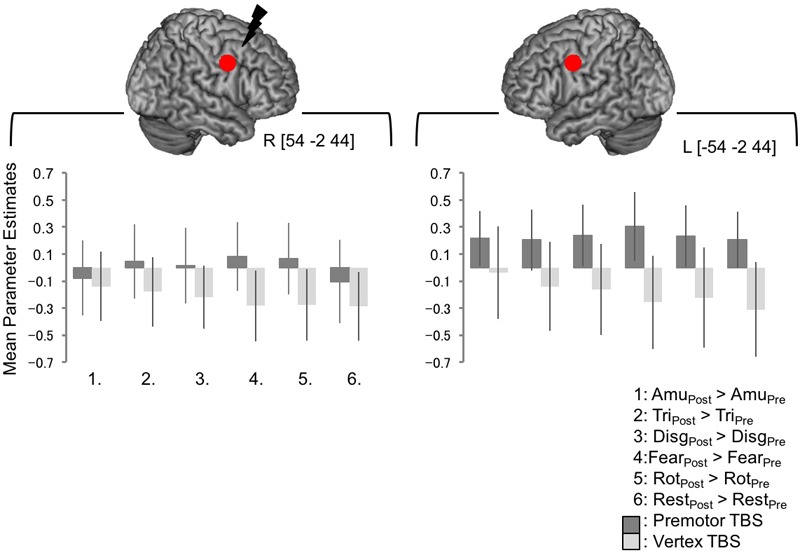
BOLD responses at the site of PMv stimulation, and the left homolog, were not significantly changed following cTBS. No significant whole brain effects were seen at the exact site of simulation or the in the corresponding site in the left hemisphere. In order to look at BOLD responses within this region further, a 6 mm spherical regions of interest were created around the coordinates the target site [54 –2 44] and the left homolog [–54 –2 44]. Mean parameter estimates were extracted and are plotted in the lower panel. The pattern of responses is not consistent across the emotional sound categories and an ANOVA indicated that there were no significant differences between any of the conditions post-stimulation compared to pre-stimulation following premotor or vertex stimulation in either site (here, a value of zero indicates no change in activity levels between pre- and post-stimulation, whereas positive values indicate greater post-stimulation activity than pre-stimulation).

Lastly, we wanted to look at BOLD responses at the target site and the left homolog of the target site. The interaction of [Session × Site] revealed a significant cluster of activity in right premotor cortex lying over the post- and precentral gyri in the right hemisphere. The peak of this cluster lay in the postcentral gyrus [66 -10 31, **Figure 3F**] with a subpeak lying at [57 2 34], around 1 cm from the target site [54 -2 44] although this cluster did not encompass precise site of targeting stimulation. In order to investigate BOLD responses within this region further, a 6 mm spherical region of interest was created around the target coordinate and the left homolog [-54 -2 44] and the mean parameter estimates were extracted for each subject. There was no significant difference between activity in this region post-vertex stimulation and post PMv stimulation (**Figure 4**).

## Discussion

A distributed network of neural regions contribute to the recognition and appraisal of affect from vocal cues (Wildgruber et al., 2005; Warren et al., 2006; Meyer et al., 2007; Paulmann et al., 2008; Wiethoff et al., 2009; Leitman et al., 2010; Ethofer et al., 2012). One component of this network is rPMv, which has been linked to passive perception of auditory emotional vocalizations (Warren et al., 2006) and the ability to discriminate between categories of vocal affect. Furthermore, cTBS targeted at rPMv results in a selective disruption in the ability to make a same/different choice on emotional vocalizations but not speaker identity (Banissy et al., 2010). By combining cTBS to the same rPMv region as Banissy et al. (2010) with fMRI, here we extend this and provide novel evidence related to the global effects of cTBS to rPMv on network wide neural activity when passively listening to affective vocal cues. Specifically, we found that cTBS to rPMv leads to secondary effects in a distributed frontoparietal network, including a rPMv region neighboring the target site, and a number of regions linked to affect appraisal and regulation. These secondary effects post rPMv stimulation were found to significantly differ to our active control stimulation site (vertex) and separated into a set of regions in which activity was increased or decreased post-rPMv stimulation. These findings therefore provide evidence that cTBS targeted at rPMv can modulate neural response to passive perception of auditory vocalizations (both emotional *and* rotated emotional vocalizations), leading to changes in widespread brain networks involved in the appraisal of affective signals.

### Increased BOLD Responses

We report that despite clear evidence to suggest that cTBS inhibits activity in the targeted region, BOLD responses were increased in a region lying close to the target site (compared to post-vertex stimulation) and across wider networks that have been linked to vocalization perception (Warren et al., 2006). While TMS pulses may inhibit a behavioral measure, the pulse ultimately results in cell firing at the site of stimulation, which may activate other neurons in distinct cortical regions (Sack and Linden, 2003). This is in contrast to our predicted findings, that cTBS would results in local inhibited activity and suppressed BOLD responses at the site of stimulation. However, this is consistent with other studies reporting that depending on task parameters, cortical suppression following brain stimulation is sometimes linked to increased BOLD response (Ruff et al., 2006; Bestmann, 2008; Ruff et al., 2008; Andoh and Zatorre, 2012) in the targeted region and vice versa (e.g., Holland et al., 2011).

Increased activity post-stimulation may reflect a rebound in cortical responsiveness following cTBS, as the site of stimulation returns to baseline levels of cortical excitability. While we did not see a significant effect at the target site, we did see a significant increase in activity in a neighboring region lying within right PMv and in a similar region in the left hemisphere. The target site, lying on the ventral bank of the boundary between PMd and PMv (Tomasino et al., 2008), contains a number of self-connections and shares connections with the homologous region in the left hemisphere, SMA, frontal regions rostral and ventral to PMv, cingulate motor areas, inferior parietal cortex/parietal operculum and posterior parietal cortex (Dancause et al., 2007). In some cases the number of connections of the PMv to the contralateral hemisphere outnumber ipsilateral connections (Dancause et al., 2007), which may explain why we see many changes to BOLD responses in the hemispheres contralateral to the site of stimulation. Alternatively, suppressing one region within the network of brain areas linked to vocalization perception may result in additional recruitment of regions across the remainder of the network in order to deal with the missing node.

The brain may actively compensate for the interference at the site of stimulation and within a wider connected network of regions (Bestmann et al., 2008; Siebner et al., 2009). Jung and Lambon Ralph (2016) showed that cTBS decreased activity at their target site, but increased activity at a contralateral site, and that activity in the right homolog region was predictive of task performance. This suggests that cTBS disruption to neural processing can lead to compensation from remote cortical regions. It has been suggested that acute adaptive reorganization following cTBS may occur in functional networks outside of the target network, which may compensate for suppression in other regions (Hartwigsen and Siebner, 2012).

Frontoparietal regions, including rPMv, are known to provide a link between the production and perception of both speech (Watkins et al., 2003; Wilson et al., 2004) and non-speech (Gazzola et al., 2006; Warren et al., 2006) articulatory and gestural actions (Ferrari et al., 2003; Gazzola et al., 2006). Several studies highlight the importance of the inferior frontal cortices in the perception and production of auditory vocalizations, and it has been suggested that the IFG comprises the final stage in processing of emotional vocalizations. Recent findings also indicate that the frontoparietal vocalization network is recruited to a greater extent when participants experience high cognitive load (McGettigan et al., 2011) or when perceiving degraded auditory stimuli (D’Ausilio et al., 2012; Kellermann et al., 2012). Continuous TBS is thought to interfere with neural function by effectively introducing noise into the neural processing in a focal region (Miniussi et al., 2010) and one interpretation is that inducing noise in rPMv results in an artificial increase in cognitive load within the network, leading to increased activity within the frontoparietal network.

### Reduced BOLD Responses Post-stimulation

In addition to the increase of activity in frontoparietal regions, a set of regions displayed the opposite pattern. These were the SMG, right IFG, cingulate cortex and two peaks within the precuneus, all of which have previously been linked with affect processing either through mechanisms of emotion perception or emotion regulation (Rankin et al., 2006; Shamay-Tsoory et al., 2009; Banissy et al., 2010; Giussani et al., 2010; Leitman et al., 2010; Lamm et al., 2011; Riem et al., 2012). The anterior cingulate is thought to form part of an attentional network serving to regulate both cognitive and emotional processing (Bush et al., 2000; Hornak et al., 2003). The caudal part of the anterior cingulate is anatomically connected to many of the regions in which activity is modified by cTBS to PMv, including parietal, premotor and supplementary motor cortex (Devinsky et al., 1995) potentially providing an anatomical link between the two networks identified here as having distinct response profiles following cTBS to PMv.

Despite previous findings showing that the effect of cTBS to PMv elicits condition specific behavioral effects, here we did not find evidence of condition specific neural effects. Specifically, that cTBS had a similar effect on BOLD responses for both emotional vocalization perception conditions, but also to spectrally rotated versions of those sounds, suggests a general effect of stimulation on auditory processing. One possibility is that cTBS to PMv serves to prime a general auditory motor network, which behaviorally modulates the processing of biologically relevant sounds, such as emotional vocalizations (Banissy et al., 2010). Other studies have demonstrated a role for right PMv in the visual processing of emotional expressions (Carr et al., 2003; Leslie et al., 2004) but also neutral expressions (Gazzola and Keysers, 2009; Caspers et al., 2010). Thus it is possible that stimulation of this ventral motor site, modulates a network that serves the sensory processing of emotional stimuli.

Given the role of each region in affect processing and potential connectivity between the two networks reported here, it is feasible that the regions showing decreased activation may serve to promote the integration and evaluation of emotion stimuli, and that suppressing rPMv results in a reduction of the functional coupling between the frontoparietal network and this set of regions. Previous work has shown that cTBS modulates functional connectivity, many showing a reduction in connectivity, both in humans (Rahnev et al., 2013; Valchev et al., 2017), and in rat brain (Mastropasqua et al., 2014), but some revealing the reverse pattern (Dan et al., 2016). In this context, prior behavioral suppression effects on vocalization discrimination seen from the application of cTBS to right PMv (Banissy et al., 2010) may originate from not only alterations in activity at the site of stimulation, but also from a reduction in a network of brain regions linked to emotion evaluation. This adds to prior concurrent TMS-fMRI work highlighting that behavioral influences of TMS may not only be a consequence of functional specialization, but also functional integration in specific tasks (Ruff et al., 2009). It also highlights cTBS-fMRI as a tool to probe the mechanisms (i.e., functional specialization vs. functional integration) by which stimulation to a target area can influence task performance.

Whilst it is clear than TMS can elicit neural plasticity and behavioral changes, there are also a range of factors that contribute to high variability in the neural and behavioral responses to stimulation, such as such as age, circadian rhythm, and endogenous brain oscillations (Ridding and Ziemann, 2010; Hamada et al., 2013). The effects of TMS on motor plasticity are known to change significantly with age (Pascual-Leone et al., 2011). It is worth considering that the effects of cTBS on the older participants in our experience may have been less than for our younger subjects. Attention is an additional factor that may be involved: in order to explicitly look at passive listening, the present study did not include an active task, however, this renders it impossible to know if subjects were paying equivalent degrees of attention across conditions or sessions. Lastly, given the number of conditions, there was relatively few trials per condition. It is possible that the paucity of condition specific effects was due to this and that further work maybe be needed to draw conclusions on how cTBS to rPMv effects specific categories of emotional processing.

## Conclusion

Previous data have shown that cTBS to the rPMv selectively disrupts the ability to perform a same/different task on emotional vocalizations; here we demonstrate that this is due to widespread changes in a network of regions involved in emotion perception and regulation. These data provide the first evidence that cTBS targeted at rPMv modulates activity in distributed cortical regions when processing emotional vocalizations. They add to previous studies (e.g., Andoh and Zatorre, 2012) by highlighting that TMS does not only influence the stimulated brain region, but extends to brain areas that are interconnected with the stimulation site. These data suggest that behavioral suppression in vocalization recognition following cTBS to rPMv (Banissy et al., 2010) reflects changes in functional interactions with interconnected cortical regions. These data therefore demonstrate the worth of using cTBS to modulate network activity when a central node is targeted and flag its utility to determine whether the behavioral effects of cTBS to a target area are due to local or distributed influences on the brain.

## Ethics Statement

This study was carried out in accordance with the recommendations of UCL Ethics Committee with written informed consent from all subjects in accordance with the Declaration of Helsinki. The protocol was approved by the UCL Ethics Committee.

## Author Contributions

ZA designed and performed the experiments, analyzed the data, and wrote the manuscript. MB designed and performed the experiments, edited the manuscript. CM carried out the experiments and edited the manuscript. VW and SS designed the experiments and edited the manuscript.

## Conflict of Interest Statement

The authors declare that the research was conducted in the absence of any commercial or financial relationships that could be construed as a potential conflict of interest.
